# Multiple intracellular signaling pathways orchestrate adipocytic differentiation of human bone marrow stromal stem cells

**DOI:** 10.1042/BSR20171252

**Published:** 2018-01-30

**Authors:** Dalia Ali, Sarah Abuelreich, Nora Alkeraishan, Najla Bin Shwish, Rimi Hamam, Moustapha Kassem, Musaad Alfayez, Abdullah Aldahmash, Nehad M. Alajez

**Affiliations:** 1 Stem Cell Unit, Department of Anatomy, College of Medicine, King Saud University, Riyadh, Kingdom of Saudi Arabia; 2 Departement de Medicine, Universite de Montreal, Montreal, Canada; 3 Molecular Endocrinology Unit (KMEB), Department of Endocrinology, University Hospital of Odense and University of Southern Denmark, Odense, Denmark; 4 Prince Naif Health Research Center, King Saud University, Riyadh, Kingdom of Saudi Arabia

**Keywords:** adipogenesis, gene expression, stem cells

## Abstract

Bone marrow adipocyte formation plays a role in bone homeostasis and whole body energy metabolism. However, the transcriptional landscape and signaling pathways associated with adipocyte lineage commitment and maturation are not fully delineated. Thus, we performed global gene expression profiling during adipocyte differentiation of human bone marrow stromal (mesenchymal) stem cells (hMSCs) and identified 2,589 up-regulated and 2,583 down-regulated mRNA transcripts. Pathway analysis on the up-regulated gene list untraveled enrichment in multiple signaling pathways including insulin receptor signaling, focal Adhesion, metapathway biotransformation, a number of metabolic pathways e.g. selenium metabolism, Benzo(a)pyrene metabolism, fatty acid, triacylglycerol, ketone body metabolism, tryptophan metabolism, and catalytic cycle of mammalian flavin-containing monooxygenase (FMOs). On the other hand, pathway analysis on the down-regulated genes revealed significant enrichment in pathways related to cell cycle regulation. Based on these data, we assessed the effect of pharmacological inhibition of FAK signaling using PF-573228, PF-562271, and InsR/IGF-1R using NVP-AEW541 and GSK-1904529A on adipocyte differentiation. hMSCs exposed to FAK or IGF-1R/InsR inhibitors exhibited fewer adipocyte formation (27–58% inhibition, *P*<0005). Concordantly, the expression of adipocyte-specific genes AP2, AdipoQ, and CEBPα was significantly reduced. On the other hand, we did not detect significant effects on cell viability as a result of FAK or IGF-1R/InsR inhibition. Our data identified FAK and insulin signaling as important intracellular signaling pathways relevant to bone marrow adipogenesis.

## Introduction

There is an increasing interest in studying the biology of bone marrow adipocytes (BMA) due to a shift in our understanding of their role as a passive filler of bone marrow space left behind during conditions of hematopoiesis impairment or bone loss [1] to an active tissue participating in bone and bone marrow homeostasis and whole body energy metabolism [2]. Regulation of BMA has been reported in a number of pathological conditions e.g. osteoporosis, glucorticoid therapy, and anorexia nervosa. Also, the biological role of BMA and their regulation seem to exhibit differences and similarities with extramedullary adipocytes [2]. Adipogenesis involves a cascade of cellular events that involve multiple intracellular signaling pathways that converge on key transcriptional factors regulating lineage commitment and differentiation (transcriptional networks and chromatin remodeling controlling adipogenesis) [3]. During commitment phase, multipotent MSCs become committed to adipocytic lineage, while in the maturation phase, MSCs are transformed into adipocytes, characterized by synthesizing and the transportation of lipid, secretion of adipocyte-specific proteins, and becoming insulin sensitive [4]. While most of the current knowledge of adipogenesis is based on studies performed on extramedullary adipocytes, a number of recent studies have focused on bone marrow adipogenesis [2,5].

In the present study, we aimed at identifying important signaling pathways that are associated with bone marrow adipogenesis based on global gene expression analysis and functional studies. We report that FAK and insulin receptor signaling pathways regulate bone marrow adipogenesis.

## Materials and methods

### Cell culture

A telomerized MSC line (hMSC-TERT) was used as a model for bone marrow-derived MSCs. This line was created through overexpression of the human telomerase reverse transcriptase gene (hTERT) and was found to express known markers of primary hMSCs and to differentiate into adipocytes and osteoblasts; the telomerization of hMSC was considered a useful model to obtain large number of cells for mechanistic studies of self-renewal and differentiation of stem cells [6,7]. These cells are referred to as hMSCs throughout this manuscript. hMSCs were maintained in Dulbecco’s modified Eagle medium (DMEM) supplemented with 10% fetal bovine serum (FBS), 1% penicillin streptomycin, and 1% nonessential amino acid (NEAA). All reagents were purchased from Gibco-Invitrogen, U.S.A. Control wells were treated with dimethyl sulfoxide (DMSO, Sigma).

### Adipocytic differentiation

hMSCs were seeded into four-well plates and exposed to adipogenic induction media composed of DMEM, 10% fetal bovine serum (FBS), 10% horse serum (Gibco, U.S.A.), 100 µM dexamethasone (Sigma, U.K.), 1 µM Rosiglitazone (BRL) (Novo Nordisk Bagsvaerd, Denmark), 3 µg/ml insulin (Sigma, U.K.), 450 µM isobutylmethylxanthine (IBMX) (Sigma, U.K.), and 1% penicillin-streptomycin (Sigma, U.K.) supplemented with FAK inhibitors (PF-573228 and PF-562271) or IGF-1R/InsR inhibitors (NVP-AEW541 and GSK1904529A), which were purchased from Selleckchem Inc. (Selleckchem Inc., Houston, TX, U.S.A.). Inhibitors were used at 5 µM throughout all experiments. Adipocyte induction medium (AIM) was changed every 2 days and for 7 days. Previous published work from our group indicated day 7 as a time point on which adipogenic markers were up-regulated significantly in an enriched population of 70–80% of adipogenic populations [8].

### Oil Red O staining

Adipocytic differentiation was determined by qualitative Oil Red O staining for lipid-filled adipocytes. Cells were washed with phosphate-buffered saline (PBS), fixed with 4% paraformaldehyde for 10 min, and then incubated with freshly made and filtered Oil Red O staining solution (0.05 g in 60% isopropanol; Sigma-Aldrich, U.S.A.) for 1 h at room temperature. Images were acquired using a Zeiss inverted microscope (ZEISS Inc., Germany).

### Nile Red quantification

Nile Red Staining assay quantification assay was performed as we previously described [9]. It is a direct stain for the detection of intracellular lipid droplets by fluorescence microscopy, flow cytofluorometry, and plate reader. It is strongly fluorescent and does not dissolve the lipids nor interact with any tissue constitutes [10]. For Nile Red Quantification, cells were cultured in polystyrene flat-bottom 96-well tissue culture-treated black microplates (Corning Inc., Corning, NY, U.S.A.). Nile Red working solution was prepared from a stock solution of 1 mg/ml. Cells were washed with PBS (Gibco, U.K.). Dye was added directly to the cells (5 μg/ml in PBS), and incubated for 10 min at room temperature in the dark, then washed twice with PBS. Fluorescent signal was measured using a SpectraMax/M5 fluorescence spectrophotometer plate reader (Molecular Devices Co., Sunnyvale, CA, U.S.A.) using bottom well scan mode, where nine readings were taken per well, using excitation of 485 nm and emission of 572 nm. Furthermore, fluorescent images were captured using a FLoid Cell Imaging Station (Life Technologies Inc., Grand Island, CA, U.S.A.).

### Total RNA isolation and real-time PCR

Total RNA was isolated from cells using a total RNA Purification kit (Norgen-Biotek Corp., Canada) according to the manufacturer’s instructions. The concentration and quality of total RNA were measured using NanoDrop 2000 (Thermo Scientific, U.S.A.). Reverse transcription was performed on 500 ng of total RNA using the High-Capacity cDNA Reverse Transcription kit (Applied Biosystem, U.S.A.). Expression levels of adipocyte-related genes [Adipocyte protein 2 (AP2), adiponectin (AdipoQ), and CCAAT-enhancer-binding protein α (CEBPα)] were quantified using the Fast SYBR Green Master Mix and the ViiA 7 Real-Time PCR device (Applied Biosystem, U.S.A.). Primers used for gene expression analysis are listed in Table 1. The 2DCT value method was used to calculate relative expression, as previously described [11].

**Table 1:** List of SYBR green primers used in the present study

No.	Name	Sequence
1	AdipoQ	F: 5′-GCAGTCTGTGGTTCTGATTCCATAC
		R: 5′-GCCCTTGAGTCGTGGTTTCC
2	FABP4	F: 5′-TGGTTGATTTTCCATCCCAT
		R: 5′-GCCAGGAATTTGACGAAGTC
3	CEBPA	F: 5′-TATAGGCTGGGCTTCCCCTT
		R: 5′-AGCTTTCTGGTGTGACTCGG
5	β-Actin	F: 5′-AGCCATGTACGTTGCTA
		R: 5′-AGTCCGCCTAGAAGCA
6	SORBS1	F: 5′-GCGACCACTGGTGAAAAACC
		R: 5′-CTGGAAAACTGTGGGCTTGC
7	CDC25B	F: 5′-GCTCTCAGTCCAGCAGGC
		R: 5′-ACTCTTTGGGGTTTCGCTGC
8	PCNA	F: 5′-GCTCTTCCCTTACGCAAGTCT
		R: 5′-TAGCTGGTTTCGGCTTCAGG
9	YWHAH	F: 5′-CCGCTATGAAGGCGGTGAC
		R: 5′-AAGATCGCCTGGCACCAAC

### Gene expression microarray

RNA isolation and gene expression analyses were carried out in accordance with our previously published protocol [12]. In brief, hMSCs were differentiated into adipocytes and on day 7, RNA was isolated using Total Tissue RNA Purification Kit from Norgen-Biotek Corp. (Thorold, ON, Canada) and were quantified using NanoDrop 2000 (Thermo Scientific, Wilmington, DE, U.S.A.). Total RNA was labeled and then hybridized to the Agilent Human SurePrint G3 Human GE 8 × 60 k mRNA microarray chip (Agilent Technologies). All microarray experiments were conducted at the Microarray Core Facility (Stem Cell Unit, Department of Anatomy, King Saud University College of Medicine). Data were subsequently imported into GeneSpring 13.0 (Agilent Technologies, Palo Alto, CA, U.S.A.) and were normalized using percentile Shift, whereas Benjamini–Hochberg False Discovery Rate (FDR) method was used for multiple testing corrections. Two-fold cutoff and *P* (corr) < 0.05 were used to determine significantly changed transcripts. Pathway analyses were conducted using the Single Experiment Pathway analysis feature in Gene Spring 13.0. Gene expression datasets were deposited to the Gene Expression Omnibus (GEO) under accession number GSE107789.

### Alamar Blue cell viability assay

Cell viability was measured using alamarBlue assay according to the manufacturer’s recommendations (AbD Serotec, Raleigh, NC, U.S.A.). In brief, we cultured cells in 96-well plates in 100 μl of the appropriate medium and at the indicated time point, and 10 μl of alamarBlue substrate was added and plates were incubated in the dark at 37°C for 1 h. Reading was subsequently taken using fluorescent mode (Ex 530 nm/Em 590 nm) using BioTek Synergy II microplate reader (BioTek Inc., Winooski, VT, U.S.A.).

### Statistical analysis

Statistical analysis and graphing were performed using Microsoft Excel 2010 and GraphPad Prism 6 software (GraphPad software, San Diego, CA, U.S.A.). Results were presented as mean ± SEM. Unpaired *T*-test was used to calculate statistical significance.

## Results

### Multiple intracellular signaling pathways are associated with bone marrow adipogenesis

**Figure 1:**
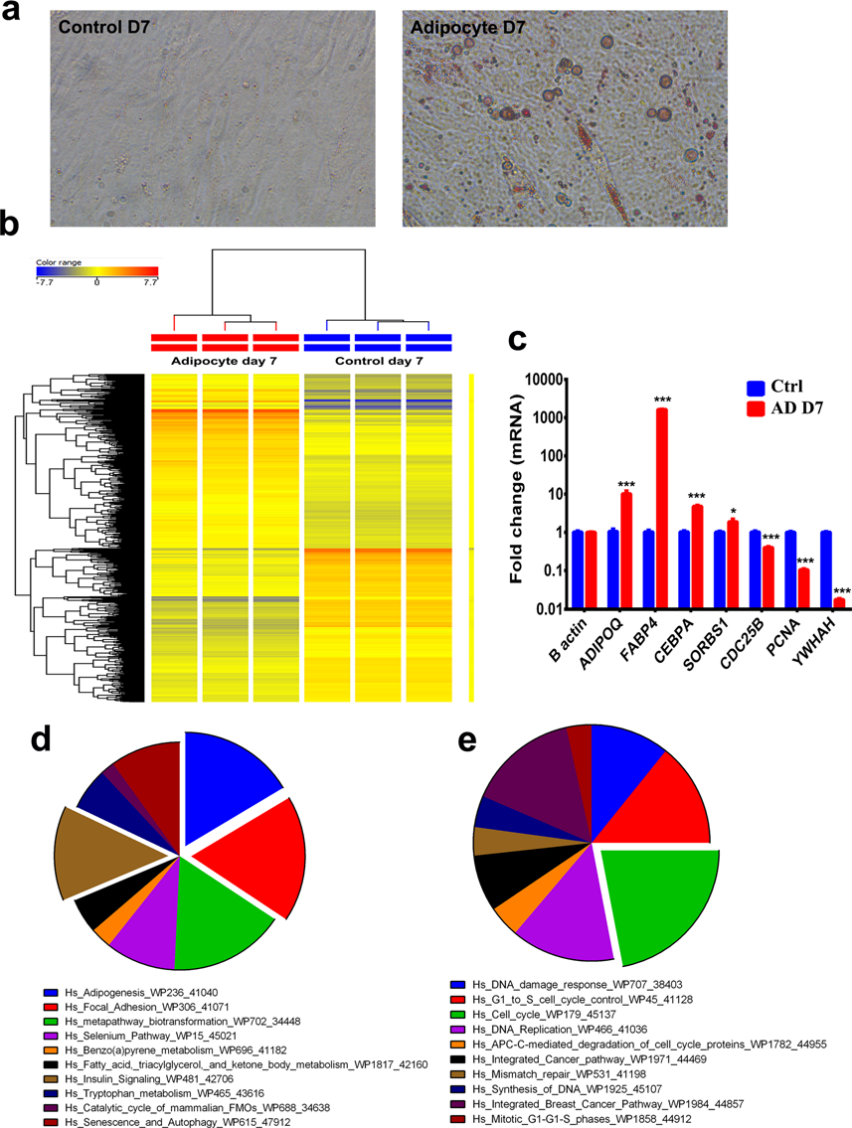
Microarray gene expression profiling of adipocyte differentiated hMSCs (**a**) Representative Oil Red O staining of lipid-filled adipocytes on day 7 for uninduced (left) or induced (right) hMSCs. (**b**) Heat map analysis and unsupervised hierarchical clustering were performed on differentially expressed genes in adipocyte day 7 vs control hMSCs. (**c**) Validation of a selected panel of up-regulated genes during adipocyte differentiation by qRT-PCR. Gene expression was normalized against β-actin. Data are presented as mean fold change ± SEM (*n*=6) from two independent experiments; *P<0.05; ***P<0.0005. (**d**) Pie chart illustrating the distribution of the top ten enriched pathway categories for the up-regulated genes identified in adipocyte day 7 vs control hMSCs. (**e**) Pie chart illustrating the distribution of the top ten enriched pathway categories for the down-regulated genes identified in adipocyte day 7 vs control hMSCs.

In order to study more in the genetic program associated with bone marrow adipogenesis, we utilized a telomerized MSC line (hMSC-TERT). This model was previously shown to express markers representative of human MSCs and to differentiation into adipocytes, osteoblasts, and chondrocytes [6,7]. Global gene expression and pathway analysis were conducted on enriched culture of BMA (70–80%) using our standard adipocytic differentiation protocol [13–15]. The efficacy of adipocyte differentiation was evidenced by formation of enriched population of mature lipid-filled adipocytes as demonstrated by positive staining for Oil Red O (Figure 1a). Global gene expression profiling revealed 2,589 up-regulated and 2,583 down-regulated mRNA transcripts during adipogenesis (Figure 1b and Supplementary Table S1). Validation of selected number of genes from the microarray data using qRT-PCR is shown in Figure 1c. Pathway analysis carried out on the up-regulated gene list revealed enrichment in multiple GO categories and signaling pathways (Supplementary Table S2) and the top ten enriched pathways included adipogenesis, Insulin Signaling, focal adhesion signaling, metapathway biotransformation, and a number of metabolic pathways: selenium metabolism, benzo(a)pyrene metabolism, fatty acid, triacylglycerol, and ketone body metabolism, tryptophan metabolism, catalytic cycle of mammalian FMOs (Figure 1d). Similarly, we performed GO enrichment and pathway analysis on the down-regulated genes, which revealed significant enrichment, in gene categories and pathways related to cell cycle (Figure 1e and Supplementary Table S3).

### Pharmacological inhibition of FAK or IGF-1R/InsR impairs adipocytic differentiation of hMSCs

**Figure 2:**
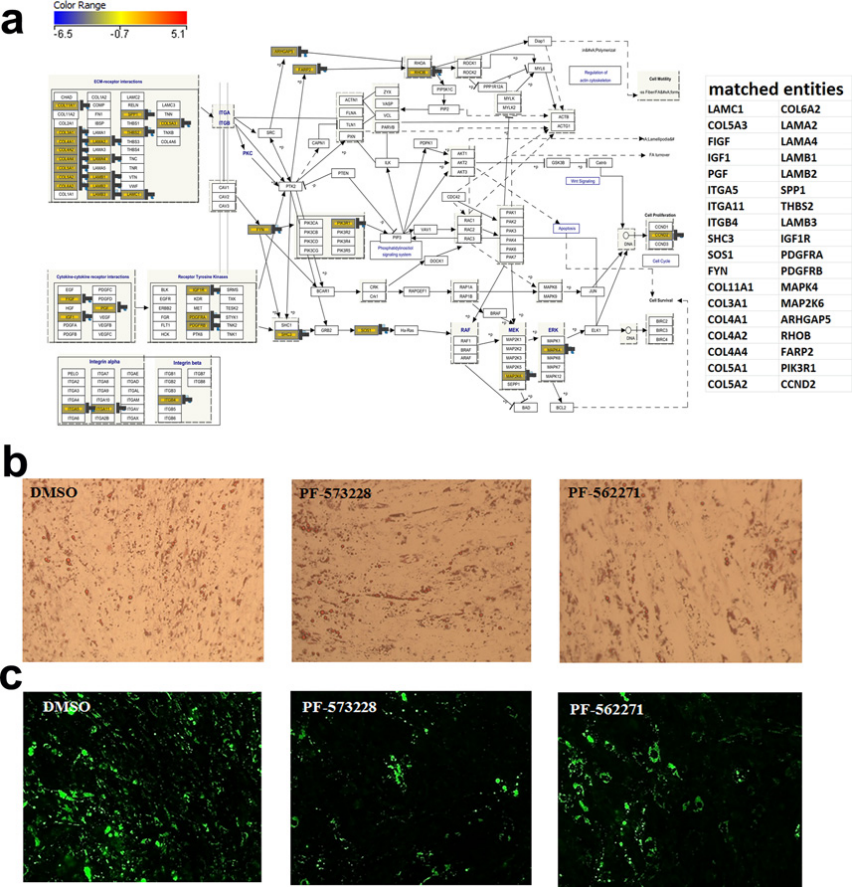
Effect of pharmacological inhibition of FAK on adipocyte differentiation (**a**) Illustration of the FAK signaling pathway with matched entities from the microarray data highlighted and listed on the right panel. hMSCs were induced into adipocytes in the absence or presence of 5 μM of PF-573228 or PF-562271 FAK inhibitors and were subsequently stained with Oil Red O (**b**) or Nile Red (**c**) on day 7. Data are representative of at least two independent experiments. Oil Red O images (10×) were acquired using an inverted Zeiss microscope, while Nile Red images were captured using FLOID imager (20×).

In order to assess the role of the identified signaling pathways on regulating BM adipogenesis, we focused on focal adhesion kinase and insulin signaling pathways and we employed small molecule inhibitors in these studies. Illustration of the focal adhesion kinase pathway is shown in Figure 2a with marking of the identified regulated genes from the microarray data. hMSCs were cultured under adipocytic conditions in the absence or presence of two FAK inhibitors (PF-573228 or PF-562271 at 5 μM) for 7 days. We used two different FAK inhibitors to confirm that the observed effect is indeed due to FAK inhibition, and not due to off-target effects of the inhibitors. Data presented in Figure 2 demonstrate reduction in the number of adipocyte formed following treatment with PF-573228 or PF-562271, compared with the DMSO control (Figure 2b) as evidenced by decreased Oil Red O staining (Figure 2b) or adipocyte cell number determined by Nile Red staining (Figures 2c and 4a).

**Figure 3:**
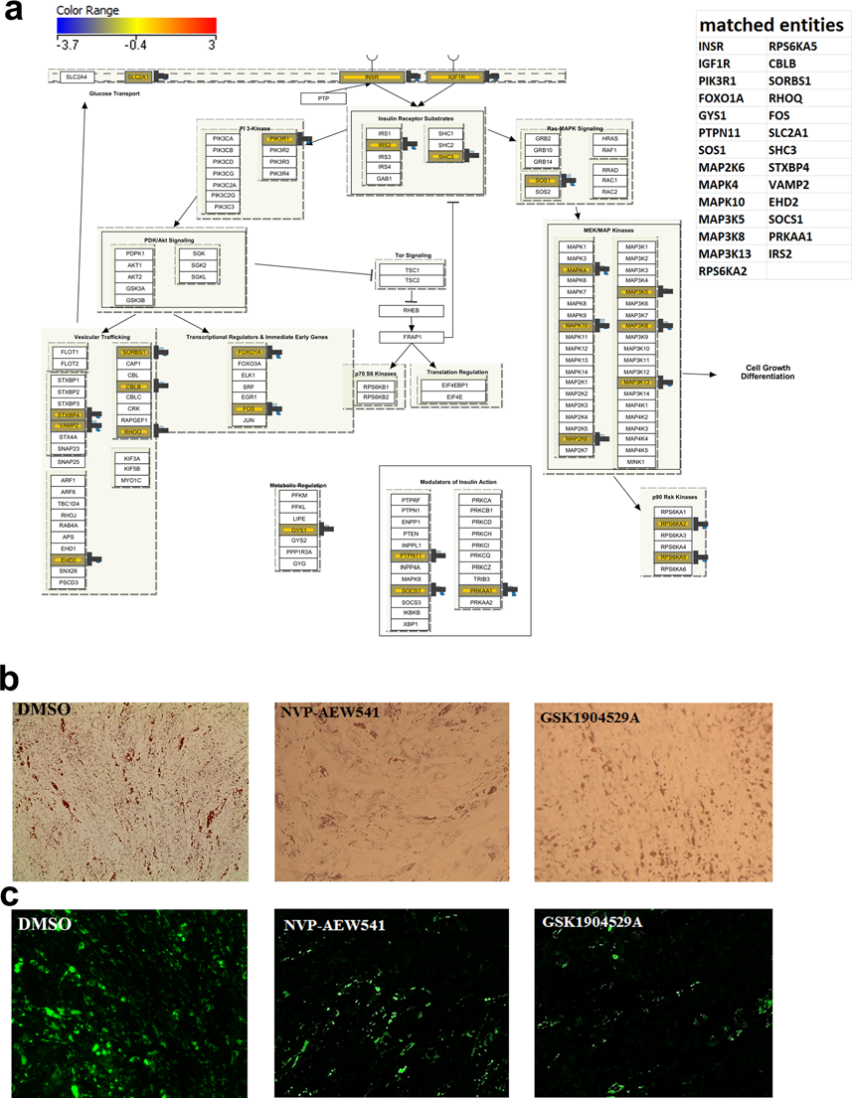
Effect of pharmacological inhibition of IGF-1R/InsR pathways on adipocyte differentiation (**a**) Illustration of the Insulin signaling pathway with matched entities from the microarray data highlighted and listed on the right panel. hMSCs were induced into adipocytes in the absence or presence of 5 μM of NVP-AEW541 or GSK1904529A IGF-1R/InsR inhibitors and were subsequently stained with Oil Red O (**b**) or Nile Red (**c**) on day 7. Data are representative of at least two independent experiments. Oil Red O images (10×) were acquired using an inverted Zeiss microscope, while Nile Red images were captured using FLOID imager (20×).

**Figure 4:**
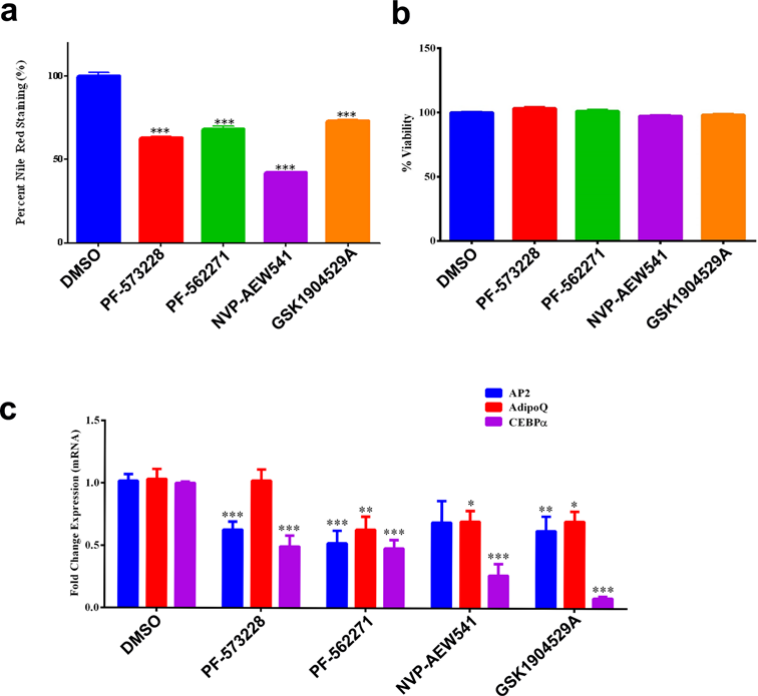
Inhibition of FAK and IGF-1R/InsR pathways reduces adipocytic-gene markers hMSCs were induced into adipocytes in the absence or presence of 5 μM of FAK (PF-573228 or PF-562271) or IGF-1R/InsR (NVP-AEW541 or GSK1904529A) inhibitor and on day 7 quantification of Nile Red fluorescence (**a**) and AlamarBlue cell viability (**b**) under different treatment conditions was performed. Data are presented as mean ± SEM from two independent experiments; *n*=12, ***P<0.0005. hMSCs were induced into adipocytes in the absence or presence of FAK or IGF-1R/InsR inhibitors as above followed by qRT-PCR analysis of AP2, AdipoQ, and CEBPα adipocytic gene markers. Gene expression was normalized against β-actin. Data are presented as mean ± SEM of fold change compared with a DMSO control; *n*=6 from two independent experiments; *P<0.05, **P<0.005, and ***P<0.0005.

We subsequently investigated the role of insulin signaling in regulating hMSC differentiation into adipocytes. Insulin signaling is illustrated in Figure 3a with marking of the genes identified regulated genes in the microarray data. As shown, significant up-regulation of insulin receptor (INSR) and the insulin-like growth factor receptor 1 (IGF1R) were observed during BM adipogenesis. Inhibition of INSR/IGF1R signaling using NVP-AEW541 or GSK1904529A at 5 μM reduced the formation of adipocytes evidenced by decreased of Oil Red O staining (Figure 3b) and the number of adipocytes stained by Nile Red (Figures 3c and 4a). To confirm that the reduction in adipocytic differentiation of hMSCs treated with FAK or IGF-1R/InsR inhibitors is due to inhibition of adipogenesis, the viability of hMSCs cultured under control DMSO or in the presence of PF-573228, PF-562271, NVP-AEW541, and GSK1904529A was assessed on day 7 using the alamarBlue assay. Inhibition of FAK or IGF-1R/InsR was found to exert no significant effects on hMSC viability (Figure 4b).

### Pharmacological inhibition of FAK or IGF-1R/InsR reduces the expression of key genes involved in BM adipogenesis

The expression of AP2, AdipoQ, and CEBPα which are adipocytic-specific genes in hMSCs in the absence or presence of FAK and IGF-1R/InsR inhibitors was assessed using qRT-PCR. Concordant with the Oil Red O and Nile Red results, significant reduction in adipocytic gene markers in hMSCs treated with PF-573228, PF-562271, NVP-AEW541, and GSK-1904529A inhibitors (*P<0.05, **P<0.005, ***P<0.0005) (Figure 4c).

## Discussion

Adipocyte differentiation is a highly controlled process characterized by two phases: commitment phase and differentiation phase. Commitment phase results in conversion of the stem cell to a preadipocytic cells, whereas in the differentiation phase, the preadipocyte acquires the characteristics of the mature adipocyte [4]. Limited information is available regarding molecular mechanisms and signaling pathways associated with bone marrow adipogenesis. In the present study, based on global gene expression profiling of bone marrow adipogenesis, we identified multiple activated signaling pathways during adipogenesis. FAK and insulin signaling were the most prominent and we studied their functional relevance using small molecule inhibitors.

Focal adhesion kinase (FAK) is a nonreceptor protein tyrosine kinase that is constitutively associated with the integrin receptor β-integrin and plays a role in cell adhesion, differentiation, and migration in several stem cell types [16,17]. The binding of integrin to the extracellular components (ECM) leads to the activation of FAK, which is demonstrated by autophosphorylation at Tyr397 [18]. When FAK is activated, it binds to PI3K, which phosphorylates PIP2 producing PIP3. PIP3 functions to activate the downstream signaling component, protein kinase Akt, which subsequently activates adipogenesis [19–21]. In present study, we observed large number of ECM proteins to be up-regulated during adipogenesis. Several of those belonged to the collagen (COL11A1, COL12A1, COL15A1, COL18A1, COL19A1, COL27A1, COL3A1, COL4A1, COL4A2, COL4A3BP, COL4A4, COL4A5, COL5A1, COL5A2, COL5A3, COL6A1, COL6A2, COL6A2, COL6A3, COL6A3, COL7A1, COL8A1, and COL8A2) and laminin (LAMA2, LAMA4, LAMB1, LAMB2, LAMB2, LAMB3, and LAMC1) families. Therefore, it is plausible that during adipocytic differentiation FAK pathway is activated as a result of preferential expression of ECM proteins, which subsequently interact with ITGA and ITGB receptors.

Concordant with our findings, the loss of the insulin/IGF-1 signaling cascade leads to inhibition of adipogenesis [22,23]. IGF-1 has been reported to regulate adipogenesis through up-regulation of adipocyte-specific transcriptional factors CEBPα [24]. A previous study has reported the regulation of adipocyte differentiation through IGF-1 receptor/Rho GTPase pathway in 3T3-L1 cells [25]. One of the downstream regulatory targets of IGF1R signaling is Rho GTPase activity known to be important for adipogenesis, with reduced Rho activity favoring adipogenesis. Rho inhibitory protein p190-B RhoGAP down-regulates Rho GTPase activity and increases IGF-1 signaling to downstream proteins involved in adipocyte differentiation. Insulin receptor substrate 1 (IRS) transmits signals from IGF-1 to the PI3K/MAPK intercellular pathways, which eventually leads to adipocyte differentiation [25]. In the present study, we observed up-regulation of both INSR and IGF1R as well as several downstream mediators of the MEK/MAPK (MAP2K6, MAP3K13, MAP3K5, MAP3K8, MAPK10, and MAPK4) pathway. Interestingly, both ITGA/ITGB and IGF1R signaling pathways converge through activation of focal adhesion kinase 1 (PTK2, Figure 2a), thus highlighting a pivotal role for this signaling pathway during adipogenesis. The decrease in number of differentiated hMSC following FAK or IGF-1R/InsR signaling may suggest inhibition of adipocyte hyperplasia but not hypertrophy. Given the similarities and differences between BMA and extramedullary adipocytes, side-by-side comparison between those two cell types in term of signaling pathways would be the subject of an independent investigation.

## Conclusions

In the present study, we highlight the involvement of two distinctive pathways known to be involved in adipogenesis differentiation, FAK and insulin signaling pathways cross-talk (Figures 2a and 3a). Genes expressed under each pathway need further investigation as they draw a roadmap and possible targets connected specifically to FAK and Insulin signaling pathway to reverse the adipogenic differentiation and potential utilization in the treatment of adipogenesis-related metabolic disorders.

## Supporting information

**Table:** 

**Table:** 

**Table:** 

## Abbreviations

AdipoQ: adiponectin
AIM: adipocyte induction medium
AP2: adipocyte protein 2
BMA: bone marrow adipocytes
CEBPα: CCAAT-enhancer-binding protein α
DMEM: Dulbecco’s Modified Eagle’s Medium
DMSO: dimethyl sulfoxide
FAK: focal adhesion kinase
FDR: false discovery rate
hMSC: human mesenchymal stem cell
hTERT: human telomerase reverse transcriptase gene
IBMX: isobutylmethylxanthine
IGF-1R: insulin-like growth factor 1 receptor
InsR: insulin receptor
PBS: phosphate-buffered saline
qRT-PCR: quantitative reverse transcription polymerase chain reaction

## References

[1] Cawthorn W.P. and Scheller E.L. (2017) Bone marrow adipose tissue: formation, function, and impact on health and disease. Front. Endocrinol 8 10.3389/fendo.2017.00112 PMC5447009 28611729

[2] Tencerova M. and Kassem M. (2016) The bone marrow-derived stromal cells: commitment and regulation of adipogenesis. Front. Endocrinol. 7, 127 27708616 10.3389/fendo.2016.00127 PMC5030474

[3] Siersbaek R., Nielsen R. and Mandrup S. (2012) Transcriptional networks and chromatin remodeling controlling adipogenesis. Trends Endocrinol. Metab. 23, 56–64 10.1016/j.tem.2011.10.001 22079269

[4] Hamam D. (2015) microRNAs as regulators of adipogenic differentiation of mesenchymal stem cells. Stem Cells Dev. 24, 417–425 10.1089/scd.2014.0331 25405998 PMC4313403

[5] Abdallah B.M. and Kassem M. (2012) New factors controlling the balance between osteoblastogenesis and adipogenesis. Bone 50, 540–545 10.1016/j.bone.2011.06.030 21745614

[6] Simonsen J.L. (2002) Telomerase expression extends the proliferative life-span and maintains the osteogenic potential of human bone marrow stromal cells. Nat. Biotechnol. 20, 592–596 10.1038/nbt0602-592 12042863

[7] Abdallah B.M. (2005) Maintenance of differentiation potential of human bone marrow mesenchymal stem cells immortalized by human telomerase reverse transcriptase gene despite [corrected] extensive proliferation. Biochem. Biophys. Res. Commun. 326, 527–538 10.1016/j.bbrc.2004.11.059 15596132

[8] Al-Nbaheen M. (2013) Human stromal (mesenchymal) stem cells from bone marrow, adipose tissue and skin exhibit differences in molecular phenotype and differentiation potential. Stem Cell Rev. 9, 32–43 10.1007/s12015-012-9365-8 22529014 PMC3563956

[9] Hamam D. (2014) microRNA-320/RUNX2 axis regulates adipocytic differentiation of human mesenchymal (skeletal) stem cells. Cell Death Dis. 5, e1499 10.1038/cddis.2014.462 25356868 PMC4237271

[10] Greenspan P., Mayer E.P. and Fowler S.D. (1985) Nile red: a selective fluorescent stain for intracellular lipid droplets. J. Cell Biol. 100, 965–973 10.1083/jcb.100.3.965 3972906 PMC2113505

[11] Al-toub M. (2015) CDH1 and IL1-beta expression dictates FAK and MAPKK-dependent cross-talk between cancer cells and human mesenchymal stem cells. Stem Cell Res. Ther. 6, 135 10.1186/s13287-015-0123-0 26204886 PMC4533790

[12] Ali D. (2017) CUDC-907 promotes bone marrow adipocytic differentiation through inhibition of histone deacetylase and regulation of cell cycle. Stem Cells Dev. 26, 353–362 10.1089/scd.2016.0183 27829312

[13] Scotti E. and Tontonoz P. (2010) Peroxisome proliferator-activated receptor gamma dances with different partners in macrophage and adipocytes. Mol. Cell Biol. 30, 2076–2077 10.1128/MCB.00171-10 20176804 PMC2863579

[14] Ntambi J.M. and Young-Cheul K. (2000) Adipocyte differentiation and gene expression. J. Nutr. 130, 3122S–3126S 11110885 10.1093/jn/130.12.3122S

[15] Cowherd R.M., Lyle R.E. and McGehee R.E. Jr (1999) Molecular regulation of adipocyte differentiation. Semin. Cell Dev. Biol. 10, 3–10 10.1006/scdb.1998.0276 10355023

[16] Cox B.D. (2006) New concepts regarding focal adhesion kinase promotion of cell migration and proliferation. J. Cell Biochem. 99, 35–52 10.1002/jcb.20956 16823799

[17] Tomakidi P. (2014) Focal adhesion kinase (FAK) perspectives in mechanobiology: implications for cell behaviour. Cell Tissue Res. 357, 515–526 10.1007/s00441-014-1945-2 24988914

[18] Xu B., Song G. and Ju Y. (2011) Effect of focal adhesion kinase on the regulation of realignment and tenogenic differentiation of human mesenchymal stem cells by mechanical stretch. Informa Healthcare 52, 373–379 10.3109/03008207.2010.541961 21401419

[19] Aubin D., Gagnon A. and Sorisky A. (2005) Phosphoinositide 3-kinase is required for human adipocyte differentiation in culture. Int. J. Obes. (Lond.) 29, 1006–1009 10.1038/sj.ijo.0802961 15852047

[20] Maiuri T., Ho J. and Stambolic V. (2010) Regulation of adipocyte differentiation by distinct subcellular pools of protein kinase B (PKB/Akt). J. Biol. Chem. 285, 15038–15047 10.1074/jbc.M110.121434 20223817 PMC2865331

[21] Yu W. (2008) Critical role of phosphoinositide 3-kinase cascade in adipogenesis of human mesenchymal stem cells. Mol. Cell Biochem. 310, 11–18 10.1007/s11010-007-9661-9 18060476

[22] Siddals K.W. (2002) IGF-binding protein-1 inhibits IGF effects on adipocyte function: implications for insulin-like actions at the adipocyte. J. Endocrinol. 174, 289–297 10.1677/joe.0.1740289 12176668

[23] MacDougald O.A. and Mandrup S. (2002) Adipogenesis: forces that tip the scales. Trends Endocrinol. Metab. 13, 5–11 10.1016/S1043-2760(01)00517-3 11750856

[24] Liu J. (2014) Insulin-like growth factor-1 and bone morphogenetic protein-2 jointly mediate prostaglandin E2-induced adipogenic differentiation of rat tendon stem cells. PLoS One 9, e85469 10.1371/journal.pone.0085469 24416413 PMC3887066

[25] Raffaella Sordella W.J., Chen G.-C., Curto M. and Settleman J. (2003) Modulation of Rho GTPase signaling regulates a switch between adipogenesis and myogenesis. Elsevier 113, 147–158 10.1016/s0092-8674(03)00271-x 12705864

